# Many faces of rationality: Implications of the great rationality debate for clinical decision‐making

**DOI:** 10.1111/jep.12788

**Published:** 2017-07-20

**Authors:** Benjamin Djulbegovic, Shira Elqayam

**Affiliations:** ^1^ Program for Comparative Effectiveness Research University of South Florida Tampa FL USA; ^2^ Department of Internal Medicine, Division of Evidence‐based Medicine, Morsani College of Medicine University of South Florida Tampa FL USA; ^3^ Department of Hematology H. Lee Moffitt Cancer Center and Research Institute Tampa FL USA; ^4^ Department of Health Outcomes and Behavior H. Lee Moffitt Cancer Center and Research Institute Tampa FL USA; ^5^ School of Applied Social Sciences, Division of Psychology De Montfort University Leicester UK

## Abstract

Given that more than 30% of healthcare costs are wasted on inappropriate care, suboptimal care is increasingly connected to the quality of medical decisions. It has been argued that personal decisions are the leading cause of death, and 80% of healthcare expenditures result from physicians' decisions. Therefore, improving healthcare necessitates improving medical decisions, ie, making decisions (more) *rational*.

Drawing on writings from The Great Rationality Debate from the fields of philosophy, economics, and psychology, we identify core ingredients of rationality commonly encountered across various theoretical models. Rationality is typically classified under umbrella of normative (addressing the question how people “should” or “ought to” make their decisions) and descriptive theories of decision‐making (which portray how people *actually* make their decisions). Normative theories of rational thought of relevance to medicine include epistemic theories that direct practice of evidence‐based medicine and expected utility theory, which provides the basis for widely used clinical decision analyses. Descriptive theories of rationality of direct relevance to medical decision‐making include bounded rationality, argumentative theory of reasoning, adaptive rationality, dual processing model of rationality, regret‐based rationality, pragmatic/substantive rationality, and meta‐rationality.

For the first time, we provide a review of wide range of theories and models of rationality. We showed that what is “rational” behaviour under one rationality theory may be irrational under the other theory. We also showed that context is of paramount importance to rationality and that no one model of rationality can possibly fit all contexts. We suggest that in context‐poor situations, such as policy decision‐making, normative theories based on expected utility informed by best research evidence may provide the optimal approach to medical decision‐making, whereas in the context‐rich circumstances other types of rationality, informed by human cognitive architecture and driven by intuition and emotions such as the aim to minimize regret, may provide better solution to the problem at hand. The choice of theory under which we operate is important as it determines both policy and our individual decision‐making.

## 1 INTRODUCTION

The United States spends nearly 18% ($2.7 trillion) of its GDP on healthcare, yet more than 30% is wasted on inappropriate care.^1^ Increasingly, suboptimal care is connected to the quality of medical decisions.^1^ It has been argued that personal decisions are the leading cause of death^2^, and that 80% of healthcare expenditures result from physicians' decisions.^3^, ^4^ Therefore, improving healthcare necessitates improving medical decisions, ie, making decisions more *rational*. The “Great Rationality Debate”^5^, ^6^—a debate about most optimal course of our reasoning, decision‐making, and actions—has permeated the fields of philosophy, economics, and psychology for decades but remains a neglected topic in clinical literature, despite of its obvious importance.

We draw on writings from these fields to identify some core ingredients of rationality (Table 1), and demonstrate its relevance to the practice of medicine. The debate about rational thought reveals a striking lack of consensus on universally accepted definition of rationality (Table 2 lists definitions of major theories of rationality and their relevance to medicine).^7^ In turn, these different definitions of rationality have profound implications on what type of decision‐making should be embraced in clinical practice. We draw on core principles from the rationality debate to outline guidelines toward selecting context‐appropriate practical prescriptive models for rational medical decision‐making.

**Table 1:** Core ingredients (“Principles”) of rationality commonly identified across theoretical models

P1: Most major theories of choice agree that rational decision‐making requires integrations of
**Benefits** (gains)
**Harms** (losses)
in order to fulfil our ***goals*** (eg, better health).
P2: It typically occurs under conditions of ***uncertainty***.
Rational approach requires reliable evidence to deal with the inherent uncertainties.
Relies on cognitive processes that allow integration of probabilities/uncertainties.
P3: Rational thinking should be informed by ***human cognitive architecture***.
composed of type 1 reasoning processes, which characterizes “old mind” (affect‐based, intuitive, fast, resource‐frugal) and type 2 processes (analytic and deliberative, consequential driven, and effortful) of “new mind”
P4: Rationality depends on the ***context*** and should respect epistemological, environmental, and computational ***constraints*** of human brains
P5: Rationality (in medicine) is closely linked to ***ethics and morality*** of our actions
requires consideration of ***utilitarian*** (society‐oriented), ***duty‐bound*** (individual‐oriented), and ***right‐based*** (autonomy, “no decision about me, without me”) ethics

Text in bold identifies core ingredients of rationality.

**Table 2:** A list of major theories and models of rationality of relevance to medical decision‐making

**Adaptive or ecological rationality** 10, 11: a descriptive theory, a variant of bounded rationality, which stipulates that human decision‐making depends on the context and environmental cues; hence, rational behaviour/decision‐making requires adaptation to environment/patient circumstances. Sometimes referred to as “**panglossian**”^5^, ^12^, the position that humans should be considered to be *a priori* rational due to optimal evolutionary processes.
Example: dominates medical practice, which relies on extrapolation of research evidence to specific patient circumstances including social context, co‐morbidities, etc
**Argumentative Theory of Reasoning** 13, 14: proposes that reason and rational thinking has evolutionary evolved with primary social function to justify one selves and convince others to be believed and gain their trust.
Example: doctors invoke evidence‐based knowledge out of sense that it would be approved by the medical community and, in doing, preserve their reputation and improve health of their patients.
**Bounded rationality** 15, 16: a descriptive theory, which posits that reflective of principle that rationality should respect epistemological, environmental and computational constraints of human brains, rational behaviour relies on **s*atisficing*** process (finding a good enough solution) instead of EUT maximizing approach. The ***heuristic approach*** to decision‐making is the mechanism of implementation of bounded rationality.^17^ Often linked to **prescriptive** models of rationality^18^ designs for improvement of human rationality informed by cognitive architecture
Example: simple fast‐and‐frugal tree using readily available clinical cues outperformed 50 variables multivariable logistic model regarding decision whether to admit the patient with chest pain to coronary care unit.^10^
**Deontic introduction theory** 19: a descriptive theory of inference from “Is” to “Ought,” implies that rationality requires integration of the evidence related to the problem at hand (“Is”) with the goals and values to decisions and actions (“Ought”), while taking context into account. See also grounded rationality.
Example: See text.
**Dual processing theories of rational thought (DPTRT)** 5: a family of theories based on the architecture of human cognition, contrasting intuitive (type 1) processes with effortful (type 2) processes. A descriptive variant of this approach is that the rational action should be coherent with formal principles of rationality as well as human intuitions about good decisions. The normative/prescriptive variant of this theory is sometimes referred to as “**meliorism**,”^5^, ^12^ the position that humans are often irrational but can be educated to be rational. According to Meliorist principles, when the goals of the genes clash with the goals of the individual (see below), the rational course of action should be dictated by the latter.
Example: physicians often adjust their recommendations based on their intuition.^20^
DPTRT can be thought of as a combination/contrast of:
**Old mind/evolutionary rationality/rationality of the genes** 21, 22: the rationality linked to evolutionarily‐instilled goals (sex, hunger, etc). Past‐oriented and relying on type 1 mechanisms, it is driven by the evolutionary past and by experiential learning.
Example: Eating chocolates when one has to reduce weight.
**New mind/individual rationality** 21, 22: the rationality linked to the goals of the individual rather than those of the genes. It is future‐oriented and relies on type 2 mechanisms, most importantly the ability to run mental simulations of future events and hypothetical situations. This is what enables humans to think consequentially and solve novel problems.
Example: use of contraceptives. The genes' goal is to self‐replicate, ie, to produce more copies of themselves. Contraceptives negate this goal while allowing humans greater individual freedom.
**Evidence‐based medicine approach to rational decision‐making** 23: a normative theory, which posits that there is a link between rationality and believing what is true [our actions and beliefs are justifiable (or, reasonable/rational) as a function of the trustworthiness of the evidence, and the extent to which we believe that evidence is determined by credible processes]. See also epistemic rationality.
Example: Clinical practice guidelines panels more readily recommend health interventions if the quality of evidence supporting such a recommendation is high.^24^
**Epistemic rationality**: the rationality based on acquisition of true/fit‐for‐purpose knowledge. Linked to new mind rationality^21^ (see also grounded rationality).
Example: evidence‐based medicine approach to decision‐making
**Grounded rationality** 25: a descriptive theory, which postulates that rationality should be judged within epistemic context, ie, what is known to a decision maker and his/her goals, and that rational course of is the one that facilitates the achievement of our goals given the context. See also pragmatic rationality.
Example: To achieve health goals, physicians typically recommend treatment with which they are familiar/know about.
**Meta‐rationality** 6 **or the master rationality motive** 26: Relies on DPTRT, which posits that rationality represents hierarchical goal integration while taking into account both emotions and reasons. It also refers to integration of so called ***thin theories of rationality*:** Theories in which the goals, context and desires of behaviour are not evaluated (as per, for example, applying EUT without taking patient's desires into account)—that is, any goal is as good as any other goal with ***Broad theories of rationality*:** Theories of rationality in which the goals and desires of the decision maker are evaluated within context and in such a way as to achieve hierarchical coherence within goals.^22^, ^27^
Example: subsumes other variants of DPTRT and is often characteristic of a “wise” physician; the approach is particularly evident in high‐stake, high‐emotional decisions such as end‐of‐life where the substantive goals about achievable health status have to be reconciled with patient/physician emotional reaction to a proposed decision.
**Normative rationality/rationality**_**2**_ 8 **/Bayesian rationality** 28 **/EUT** 29: the type of rationality associated with conformity to a normative standard such as the probability calculus or classical logic. In medicine, the most dominant normative theory is EUT, which is based on mathematical axioms of rationality according to which rational choice is associated with selection of the alternative with higher expected utility (expected utility is the average of all possible results weighted by their corresponding probabilities). It is typically based on Bayesian probability calculus.^a^
Example: decision analyses such as EUT‐based micro simulation model to develop screening recommendations for colorectal cancer^30^
**Pragmatic/instrumental rationality/rationality**_**1**_ 8 **or substantive rationality** 31, 32: a descriptive theory, which states that rationality depends on the content not only on the structure of decisions (process) and that the content should be assessed in light of short‐ and long‐term goals (purpose). Fits with the **descriptivist** approach^9^ which argues that empirical evidence cannot support the “oughtness” of a model
Example: dominates clinical decision‐making particularly in the fields such as oncology, where desirable health goals (eg, cure) may not be possible; as a result, the re‐evaluation of both goals and decision procedures may be needed (eg, switch from aggressive treatment to palliative care in advanced incurable cancers, etc)
**Regret regulation‐rationality is characterized by regulation of regret** 33: a variant of DPTRT that relies on regret, which as a cognitive emotion uses counterfactual reasoning processes to tap in analytical aspect of our cognitive architecture as well as in affect‐based decision‐making. According to this view, medical rational decision‐making is associated with regret‐averse decision processes.
Example: Contemporary medical practice has increasingly adopted that patients' values and preferences should be consulted before a health intervention is given. However, patient values and preferences heavily depend on emotions such as regret, which, if properly elicited, may improve vigilance in decision‐making.^34^, ^35^, ^36^
**Robust satisficing** 31, 32: a variant of regret‐based DPTRT according to which rational course is to “maximize confidence in a good enough outcome even if the things go poorly” (instead of maximizing EUT); the concept, which is similar to “acceptable regret”^37^, ^38^ hypothesis of rational decision‐making, which postulates that we can rationally accept some losses without feeling regret.
Example: Annual screening mammography over 10 years in women older than 50 will prevent 1 death per 1000 from breast cancer but at cost of 50 to 200 unnecessary false alarms and 2 to 10 unnecessary breast removals.^39^ When it comes to the decisions like these, which are value‐ and emotion‐driven decisions, there is no right or wrong answers. Some women will accept harms for a small chance of avoiding death from breast cancer. Others may not.^40^
**Threshold model of rational action** proposes that the most rational decision is to prescribe treatment or order a diagnostic test when the expected treatment benefit outweighs its expected harms at given probability of disease or clinical outcome.^41^ It has been formulated both within EUT,^42^, ^43^ dual processing theories^44^ and regret framework.^37^, ^38^, ^41^, ^45^
Example: See text.

Abbreviation: EUT, expected utility theory.

Text in bold refers to headings i.e., listing of theories rationality.

a Recently classical Bayesian models were contrasted against quantum models of rationality,^46^ but at this time, the applied value of the quantum models remains uncertain.

But, first, what do we mean by “rational” medical decision‐making? Importantly, rationality does not guarantee that every single decision would be error‐free; on the contrary, rational decision‐making takes into account the consequences of possible errors—false negatives and false positives—to aid in arriving at desirable outcomes. Rationality is often defined as acting in a way that helps us achieve our *goals*,^5^, ^8^ which in clinical setting typically means desire to improve our health. Most major theories of choice agree that our goals are best achieved if we take into account both benefits (gains) and harms (losses) of alternative courses of actions, which in medical context (as in everyday life) often occur under conditions of uncertainty (principles 1 and 2, Table 1).

The “rationality debate” has revolved around the most optimal procedures that are needed to achieve our goals. This is sometimes referred to *rationality*_*1*_ *,* or *pragmatic* rationality, which *describes* what people *actually* do to achieve their goals; in contrast to *rationality*_*2*_, or *normative* rationality, which is the rationality defined by conformity to a normative standard such as expected utility theory (EUT).^8^ Even though the relation between normative and pragmatic rationalities continue to be debated, as well as the question whether normative models are necessary at all,^9^ a number of the insights that have emerged over the last several decades are already highly relevant to practice of medicine as we outline below.

To highlight the concepts we just introduced, we start with a description of the normative view of rationality in clinical medicine, mainly EUT and evidence‐based medicine (EBM); we then outline pertinent descriptive principles which normative models disregard, highlighting the importance of dual processing cognitive architecture, emotion, intuition, and context. Note that there are many decision theories; each theory has its proponents and critics—our goal is not to critically appraise the pro and con arguments of all theories of rational choice but to simply enlist common rationality theories and their potential relevance to medicine. However, we propose that dual processing architecture is a key principle of ethical medical decision‐making. We conclude by suggesting that no single model of rationality can possibly fit all contexts. We propose a prescriptive set of cognitively informed guidelines for selection of pragmatically rational behaviours for medical decision‐making, illustrated through 1 specific (ie, threshold) model.

## 2 THE NORMATIVE APPROACH TO RATIONALITY IN CLINICAL MEDICINE

Standard normative theories of rational choice rely on mathematical analyses to help derive the optimal course of action, the one which we “should” or “ought” to pursue. They typically use EUT, the basis of applied decision analysis. Importantly, EUT is the arguably only theory of choice that satisfies all mathematical axioms of rational decision‐making. According to EUT, rational decision‐making is associated with selection of the alternative with higher expected utility such as higher quality‐adjusted life years. Expected utility theory dominates medical decision‐making literature, as evidenced by numerous decision analyses aiming to generate best advice for physicians and patients. For example, the US Preventive Services Task Force used decision analysis to identify the optimal test for colorectal cancer screening.^30^

Normative theories of choice have also pointed out that rationality of choice is a matter of the procedure of choosing and not of what is chosen: A good decision can result in bad outcomes; a bad decision can result in good outcomes. However, in a long run, better decisions should also result on the whole in better outcomes. Within framework of EBM, the higher quality of evidence inspires greater confidence in the estimate of health interventions' effects, because such estimates about effects of health interventions are closer to the truth.^23^ According to this view, rationality is consistent with response to evidence in the fitting way.^23^, ^47^ All things equal, it is more rational to act based on well‐conducted randomized trials than on the observational evidence.^23^ The importance of understanding this aspect of rationality can be best appreciated within the recent US health law linking financing of healthcare with performance and “value.” Fifty per cent of physicians' compensation will be linked to the “quality” of care, which is measured by both process of care (eg, adherence to practice guidelines) and patient outcomes,^48^ although the latter are beyond physicians' control, making such a policy hardly rational. Nevertheless, EBM is yet to develop a coherent theory of healthcare decision‐making, and examples abound how evidence alone is not sufficient for effective decision‐making.^49^

Normative models of decision‐making are mathematical abstracts and must be justified philosophically and mathematically, which makes them impervious to empirical evidence of cognitive mechanisms (principle 3, Table 1). It is *prescriptive* models that bridge between normative models and human cognition and between descriptive and normative theories of rational choice. Prescriptive models are pragmatic “ought” models, sets of cognitive tools engineered to support rational decision‐making.^50^, ^51^, ^52^ As an applied field, medical decision makers face decisions fraught with moral and pragmatic significance. Developing prescriptive models of rational and ethical medical decision‐making is crucial in a way which goes beyond canned rationality in the lab.^9^ Unlike normative models, prescriptive models need to be informed by psychological evidence of the way that people actually reason and make decisions, to which we turn next.

## 3 DESCRIPTIVE PRINCIPLES OF DECISION‐MAKING I: DUAL PROCESSING, INTUITION AND EMOTION

Researchers have extensively documented the “normative‐descriptive gap”: People often violate normative standards,^27^ in particular the precepts of EUT. Descriptive theories of decision‐making attempt to explain this gap (theories of “is” versus “ought”). Two fundamental issues have been identified: Making the full computations required for EUT requires effortful analytic processes, neglecting other aspects of human cognitive architecture such as intuition and emotions stipulated by dual processing theories of human cognition (principle 3, Table 1). Dual process theories of cognition portray human cognitive architecture as composed of type 1 processes, characterized as “old mind” (affect‐based, intuitive, fast, and resource‐frugal) and type 2 processes (analytic, deliberative, consequential, and effortful) of “new mind.”^5^, ^21^, ^53^

Moreover, EUT does not (cannot and must not) take into account context or individual differences, all of which characterizes human decision‐making. As a result, people often do not calibrate well probabilities of events (eg, disease outcomes) and give much higher weight to both low probabilities (possibility effect) and high probabilities (certainty effect).^54^, ^55^, ^56^ The latter illustrates our intolerance of uncertainty and results in our “stubborn quest for diagnostic certainty,”^57^ the tendency to perform diagnostic tests even when their utility is questionable, which continues to be one of the main drivers of excessive testing and healthcare costs. Importantly, although acting according to descriptive theories violate precepts of EUT, as argued in this paper, depending on the setting, their use may help us achieve our goals better than EUT.

To understand decision‐making processes, we crucially need to understand the role emotions play when we weigh benefits and risks of our actions.^58^ The philosopher David Hume famously observed that “reason is, and ought only to be the slave of the passions”^59^—without emotion we have no goals, and without goals there is no rationality.^21^ In fact, regulation of emotions, chief among which is regret, represents one of the key ingredients of rational behaviour.^33^

Rationality often aims to regulate regret.^6^, ^31^, ^33^ We are regret‐averse: Many of our decisions are driven by desire to avoid regret and minimize risks. Regret operates via “robust satisficing”^31^, ^32^, ^33^—the concept similar to “acceptable regret”: We can rationally accept some losses without feeling regret^37^, ^38^ (Table 2). The latter is widely documented in clinical medicine where, for example, ordering myriad of tests is often acceptable practice even it deviates from the normative standards.^37^, ^38^ Regret is a cognitive emotion, characterized by a counterfactual reasoning process: We regret when we compare the actual outcome to what might have happened but did not happen. It is a powerfully aversive emotion, and we are motivated to behave in such a way so as we would not come to regret our actions. It is also a feat of effortful processing, since counterfactual thinking requires hypothetical simulation of possibilities—in other words, new mind processing. Thus, regret serves as a link between intuitive and effortful processes (Table 2) providing mechanism for dual process rationality model.^60^ When regret is taken into account, “stubborn quest for diagnostic certainty”^57^ may not be irrational.^37^, ^38^, ^61^

## 4 DESCRIPTIVE PRINCIPLES OF DECISION‐MAKING II: SATISFICING AND THE IMPORTANCE OF CONTEXT

Human processing is often too limited to find the most optimal solution to a given problem (principle 4, Table 1). Difficulties vary as a function of factors such characteristics of the decision itself (eg, high stakes vs low‐stakes situations, etc), situation/context (eg, clinical setting, time pressure, cognitive load, framing effect, social context, conflict of interest, etc), and individual characteristics of the decision maker (eg, cultural background,^12^ professional background, cognitive ability, decision‐making styles, etc).^62^ This means that rational behaviour requires adaptation to environment (adaptive or ecological rationality),^10^ and to individual characteristics (grounded rationality)^25^ (see Table 2). Because finding the optimum solution to a given problem can be resource‐ and computationally intensive, adaptive behaviours typically relies on satisficing (finding a good enough solution), as a rationality principle (bounded rationality), rather than optimizing/maximizing (striving to find a “perfect” solution).^63^ Mental shortcuts or heuristics—a “strategy that ignores part of the information, with the goal of making decisions more quickly, frugally, and/or accurately than more complex methods”^11^—rely on the satisficing principle and can sometimes outperform complex statistical models (“less‐is‐more”).^10^ That is, heuristics represent mechanisms to implement bounded rationality.^17^ Use of heuristics dominates medical teaching including wide popularity of clinical pathways and algorithms that resembles fast‐and‐frugal trees (FFT), highly effective, simple decision trees composed of sequentially ordered cues (tests) and binary (yes/no) decisions formulated via series of if‐then statements.^64^ The FFT can be linked to EUT and regret via the threshold model,^64^ which is intimately related to the question of rational decision‐making; the threshold model has been formulated both within EUT,^42^, ^43^ dual processing theories^44^ and regret framework.^37^, ^38^, ^41^, ^45^ The threshold represents a linchpin between evidence (which exists on the continuum of credibility) and decision‐making (which is a categorical exercise—we decide to act or not act).^41^

According to the EUT threshold, the most rational decision is to prescribe treatment when the expected treatment benefit outweighs its expected harms at given probability of disease or clinical outcome, regardless of context.^42^, ^43^ The EUT threshold model stipulates that as the therapeutic benefit/harm ratio increases, the threshold probability at which treatment is justified, is lowered.^42^, ^43^ Conversely, when a treatment's benefit/harm ratio is smaller, the required threshold for therapeutic action will be higher.^42^, ^43^ For example, benefit/harm ratio of administering antituberculotics to a patient with suspected tuberculosis is about 33 in terms of morbidity/mortality outcomes; according to EUT, rational physicians should then prescribe antituberculotics when the probability of tuberculosis exceeds 3% only.^65^ However, physicians would typically not treat a patient suspected of tuberculosis below a threshold between 20% and 50% largely because regret of commission associated with unnecessary treatment outweighs regret of omission due to failure to administer antituberculotics.^65^ In contrast, from the regret threshold model perspective, the most rational decision to prescribe treatment is when regret of failing to administer beneficial treatment outweighs regret of harms of unnecessary treatment.^41^ Thus, EUT and regret thresholds are often different, and might vary individually, which can explain variation in care widely documented in the contemporary practice: Physicians act as if they have different thresholds depending on context, which in turn could be a function of regret, different ways of cognitively assessing disease probability or the consequences (benefits and harms) of treatments.^41^, ^44^, ^45^ Because most evaluation of drugs effects goes through the regulatory approval agencies such as FDA, they will be approved for the use in practice only if benefits outweigh harms; similarly, most tests are perceived as harmless. This means that the threshold for prescribing drugs or ordering diagnostic tests will be predictably low according to EUT; even if that is most rational thing to do, most patients who received treatment will not actually have the disease for which treatment is given.^41^, ^45^, ^66^ This creates a paradox: EUT, the normative theory widely accepted as gold rationality standard in medicine will predictably lead to further increase (and waste) in the use of diagnostic and treatment interventions!^41^, ^45^, ^61^ Thus, insisting on applying EUT can be pragmatically irrational.

## 5 RATIONALITY AND MORALITY IN MEDICAL DECISION‐MAKING: THE CASE OF GOALS AND VALUES

Principle 5 (Table 1) states that rationality in medicine is closely linked to *ethics* and *morality* of our actions. These actions normally require consideration of all major ethical frameworks: utilitarian (society‐oriented) considerations, which emphasize the consequences of actions; and various deontological considerations, which rely on rights and duties: duty‐bound (individual‐oriented) and right‐based ethics (“no decision about me, without me” is heavily promoted within a framework of EBM). (While policy makers might prefer to communicate primarily deontological consideration,^67^ often ethical decision‐making requires both, especially in clinical medicine, where goals and consequences of actions play a major role.) Decision‐making according to each of these views focuses on somewhat different goals—interest of individual vs society, which often result in goal conflicts. For example, the recent call for person‐centred healthcare can be regarded as a shift from “medicine of the disease” goal to “medicine of the whole person” goal.^68^, ^69^ How to make rational and moral decisions when these considerations are in conflict? One of our aims is to develop guidelines for medical pragmatic rationality, the rationality focused on achieving one's goals. But goals are notoriously slippery and prone to conflict;^53^ where there is a clash between goals, pragmatic rationality faces a particular challenge.

This is a pertinent issue because uncertainty remains inherent in medical decision‐making; we cannot escape making false negative or false positive decision errors, which will affect different people in different ways.^70^ For example, attempts to reduce overtesting (false positive decisions) are beneficial to society but may lead to underuse (false negative decisions) in individual patients.^70^ Such goal conflict under conditions of irreducible uncertainty, leading to inevitable error, will generate unavoidable injustice (resulting in the trade‐off of goals of individuals vs society).^71^

Deontic Introduction Theory—a descriptive theory of inference from “is” to “ought”^19^—demonstrated that people are disposed to create novel normative rules when conditional relations (“If you smoke, you will probably get lung cancer”) causally link action and a valued (positive or negative) goal (“Lung cancer is undesirable outcome”). Value transference from outcome to action (“Smoking is bad”) and deontic bridging to an appropriate deontic operator (such as “should,” “may,” “must,” etc) result in normative (deontic) conclusions such as “You should not smoke.”^19^ Interestingly, both tendency to rely on reliable information (“Is”)^72^ and generate “ought” or “should” statements (“Faced with the knowledge that there *are* hungry children in Somalia, we easily and naturally infer that we *ought* to donate to famine relief charities”) seem to be evolutionary determined.^19^

Both rationality and morality have been portrayed as a function of the interactions between these 2 types of processes,^8^, ^73^ with utilitarian moral judgments linked to effortful processing and deontological moral judgement linked to intuitive processing. In the acute, life‐threatening situations, where avoiding harms is most important, it makes rational and moral sense to use type 1 processes, while type 2 processes may fit long‐term goals better. When the goals conflict, immediate personal goals or strong utilitarian or deontological rules can suppress normative conclusions.^19^ These processes increasingly operate in clinical setting: With rising costs in healthcare, cost‐effectiveness considerations—what is rational for society based on EUT may not be rational for individuals—cannot be avoided.^74^

Stanovich^5^, ^6^, ^27^ proposed that rationality reflects the goal integration (meta‐rationality). We should aim to coherently integrate our hierarchy of goals (“desire to act in accordance with reasons”).^6^ Both emotions and expected utility of outcomes matter.^6^ The trick may be to ask deliberatively and reflectively^75^ about the appropriateness of our emotional reactions to a decision and “to value formal principles of rationality, but not to take them too seriously.”^6^, ^32^, ^76^

## 6 TOWARD CONTEXTUALIZED RATIONAL CLINICAL DECISION‐MAKING: INTEGRATING THE CORE PRINCIPLES

Our goals in this paper were to provide a synopsis of the state of the art in the rationality debate in the contest of clinical medicine, describe some potential limitations of normative models in specific contexts of medical decision‐making, and outline guidelines toward selecting context‐appropriate prescriptive models for pragmatically rational medical decision‐making. For the first time, we provide a review of wide range of theories and models of rationality (Table 2). Contrary to the received view in much of medical decision‐making, we argue that the great rationality debate amply demonstrates that there is no one uniformly accepted way to exercise rational decision‐making. Moreover, the normative gold standard of rational decision‐making, EUT, can lead to instrumentally irrational behaviours, most worryingly overtesting and overtreatment.

We do not propose a single normative model to replace EUT; instead, we posit that unified, one‐size‐fits‐all theory of rationality may not, in fact, be possible. Fundamentally, we argue that what is “rational” behaviour under one rationality theory may be irrational under the other theory. The choice of theory under which we operate is important as it determines both policy and our individual decision‐making. In fact, we contend, we should pragmatically adopt definition of rationality to the problem at hand. Because such adoption is context‐sensitive, it is not possible to recommend any specific model. Instead, we propose that the core principles in Table 1 can potentially provide guidelines toward selecting normatively and cognitively informed prescriptive models applicable to each context. Thus, we propose that pragmatically rational medical decision‐making crucially depends on integration of the evidence related to the problem at hand (“Is”) with the goals and values to decisions and actions (“Ought”),^19^ while taking context into account. Hence, rational medical action should respect underlying evidence and be coherent with [a tractable form of] formal principles of rationality as well as human emotions and intuitions about good decisions and ethical principles taking the utility of society and the individual into account. The rejection of a unified theory of rational medical decision‐making and highlighting the need to match rationality model to specific medical circumstances has not been attempted before. Respecting the principles outlined in Table 1, one proposal for clinical medicine may play out along the following lines.

From policy perspectives, using EUT informed by best available current evidence (EBM) might be the most rational approach to decision‐making. This is because policy decisions are typically high‐level decisions voided of the granular, contextual details that characterize individual decision‐making at bedside and because policy makers are in position to draw on the necessary extensive computational resources. For example, using EUT‐based rationality USPSTF “recommends screening for colorectal cancer using fecal occult blood testing, sigmoidoscopy, or colonoscopy in adults, beginning at age 50 years and continuing until age 75 years.”^30^

This recommendation, however, applies to imaginary “average” person and needs to be tempered on the ground by contextualized adaptive and grounded rationality. For example, the patient who is chronologically older than 75 years, may actually be clinically and biologically in better condition than the patient who is nominally 75 years old. Thus, a strategy may take a form of heuristics such as relying on FFT (eg, “if the patient does not have any comorbidities and has excellent performance status, h/she should be recommended colonoscopy even if older than 75”). In other situations, acceptable regret^37^/robust satisficing^31^ may be appropriate decision‐making strategy (see Table 2).

In the end, “ practical wisdom may be the hallmark of rationality,”^31^, ^32^ often in medicine captured in terms of saying: “A good doctor knows *how* to treat/order a diagnostic test, a better one knows *when* to treat/order a test, but best one knows *when not* to do it….” The characteristics of such decision‐making, among other things are ability to distinguish between reducible and irreducible uncertainty and knowing how to handle false‐positive and false‐negative errors in explicit and transparent ways, to specify what values are placed on these errors, and to understand the potential for unavoidable injustice, because the consequences of actions may affect different individuals in different ways.^71^ We conclude that no “one size” rationality model fits all clinical circumstances and decision makers. Empirical research is needed to identify the situations which can be best matched to given rationality strategy.

## COMPETING INTERESTS STATEMENT

We declare no competing interest in relation to the subject of this manuscript.

## CONTRIBUTION

BD wrote first draft, which was revised by SE. The final version of the manuscript was agreed upon by both authors.
